# The prevalence and ingredient cost of chronic comorbidity in the Irish elderly population with medication treated type 2 diabetes: A retrospective cross-sectional study using a national pharmacy claims database

**DOI:** 10.1186/1472-6963-13-23

**Published:** 2013-01-16

**Authors:** Miriam O’Shea, Mary Teeling, Kathleen Bennett

**Affiliations:** 1 Department of Pharmacology and Therapeutics, Trinity Centre for Health Sciences, St James’s Hospital, James’s Street, Dublin 8, Ireland

**Keywords:** Comorbidity, Type 2 diabetes, Pharmacoepidemiology, RxRisk V index, Ireland, Ingredient cost, Elderly

## Abstract

**Background:**

Comorbidity in patients with diabetes is associated with poorer health and increased cost. The aim of this study was to investigate the prevalence and ingredient cost of comorbidity in patients ≥ 65 years with and without medication treated type 2 diabetes using a national pharmacy claims database.

**Methods:**

The Irish Health Service Executive Primary Care Reimbursement Service pharmacy claims database, which includes all prescribing to individuals covered by the General Medical Services scheme, was used to identify the study population (≥ 65 years). Patients with medication treated type 2 diabetes (T2DM) were identified using the prescription of oral anti-hyperglycaemic agents alone or in combination with insulin as a proxy for disease diagnosis. The prevalence and ingredient prescribing cost of treated chronic comorbidity in the study population with and without medication treated T2DM were ascertained using a modified version of the RxRiskV index, a prescription based comorbidity index. The association between T2DM and comorbid conditions was assessed using logistic regression adjusting for age and sex. Bootstrapping was used to ascertain the mean annual ingredient cost of treated comorbidity. Statistical significance at p < 0.05 was assumed.

**Results:**

In 2010, 43165 of 445180 GMS eligible individuals (9.7%) were identified as having received medication for T2DM. The median number of comorbid conditions was significantly higher in those with T2DM compared to without (median 5 vs. 3 respectively; p < 0.001). Individuals with T2DM were more likely to have ≥ 5 comorbidities when compared to those without (OR = 2.82, 95% CI = 2.76-2.88, p < 0.0001). The mean annual ingredient cost for comorbidity was higher in the study population with T2DM (€1238.67, 95% CI = €1238.20 - €1239.14) compared to those without the condition (€799.28, 95% CI = €799.14 - € 799.41).

**Conclusions:**

Individuals with T2DM were more likely to have a higher number of treated comorbid conditions than those without and this was associated with higher ingredient costs. This has important policy and economic consequences for the planning and provision of future health services in Ireland, given the expected increase in T2DM and other chronic conditions.

## Background

Diabetes is increasingly being recognised as a major global health concern [1]. The prevalence of diabetes has increased globally [2] and it is projected that worldwide the prevalence will increase from an estimated 2.8% in the year 2000 to 4.8% by 2030 [3]. This increase has been attributed to a rise in the incidence of type 2 diabetes (T2DM) [1], the most common type of diabetes [4], and has been driven primarily by increasing levels of obesity, inactivity and population aging [1].

Comorbidity, the co-existence of one or more additional conditions in persons with a specified index medical condition [5], is highly prevalent in patients with diabetes [6]. In the United States the majority of adults with diabetes have more than one comorbid condition [6] and 40% have 3 or more conditions [7]. A number of different frameworks have been developed that categorise comorbid conditions according to their influence on the clinical management of the index condition [8]. The most recent was developed by Piette & Kerr [9] and has since been used in a number of published studies [10-12]. The original paper classified chronic comorbid conditions as being either concordant or discordant with diabetes. Concordant conditions are those associated with diabetes, i.e. they represent part of the same overall pathophysiological risk profile and share the same medical management plan (e.g. hypertension, ischemic heart disease, and hyperlipidemia) [9,10]. In contrast, discordant conditions are not pathophysiologically associated with diabetes and consequently their management may be different (e.g. arthritis, chronic obstructive pulmonary disease, depression) [9,10].

In terms of the patient, comorbidity is associated with reduced health status, decreased quality of life and increased risk of mortality [5]. Patients with diabetes are required to manage their condition in order to obtain and maintain optimal outcome measures [13]. Depending on the symptoms and severity of the comorbid condition(s) present, patients’ prioritisation and self-management may be compromised and/or complicated [14,15]. Patients with multiple conditions may encounter conflicting medical advice and fragmented care pathways which may provide a barrier to effective self-management [15,16]. The management of comorbid conditions may also indirectly affect diabetes self care by representing an additional demand on patient time, effort and financial resources [9].

In terms of the health service, comorbidity is associated with increased health care utilisation [17] and economic cost [18-21]. In the Irish CODEIRE study, which estimated the economic cost of diabetes in Ireland, complications related to diabetes accounted for the majority (61.7%) of all patient costs [22]. This is consistent with the results of previous research conducted in Europe [23]. However, these studies did not include any cost analysis relating to discordant comorbidity.

It is evident from the published literature, that comorbidity has substantial implications in terms of self care and health service provision. At present, few Irish studies have examined the subject of comorbidity in relation to diabetes. Those that have been published have concentrated on the prevalence or impact of a single comorbid condition [24-26]. The aim of this study was to investigate and estimate the prevalence, type and ingredient cost of chronic comorbid conditions occurring in elderly Irish individuals with T2DM, compared to those without T2DM, using a modified version of the RxRisk V comorbidity index, based on data obtained from a national pharmacy claims database.

## Methods

A retrospective cross-sectional study, utilising the Irish Health Service Executive Primary Care Reimbursement Service (HSE–PCRS) national pharmacy claims database was conducted using data from 2010. The HSE-PCRS database is used primarily to provide financial reimbursement to health care professionals involved in primary care, for the provision of health services and prescription medications under a number of different state provided health care schemes, including the General Medical Services scheme (GMS) [27]. The GMS scheme provides eligible individuals, termed “medical card” patients, with access to free health care, routine dental services and prescription medication [27]. Eligibility for the GMS scheme is based on an individual being ordinarily resident in Ireland and the outcome of a gross income means assessment. The weekly income threshold for GMS scheme eligibility is dependent on the marital status and age of the claimant [27]. Older individuals aged ≥ 70 years were automatically entitled to a medical card regardless of their income from July 2001 – Dec 2008 [27]. In 2010 the GMS scheme covered half (50.4%) of the Irish population aged between 65–69 years and 98.4% of the population aged ≥ 70 years or more (based on population estimates) [28,29].

The HSE-PCRS collates information on dispensed prescribed medication for the GMS scheme on a monthly basis. Medications dispensed through the GMS scheme are recorded in the HSE-PCRS and are coded using the WHO Anatomical Therapeutic Chemical (ATC) classification system. In addition to providing details on medications dispensed to eligible individuals, the HSE-PCRS pharmacy claims database also contains demographic information about the claimant such as age, sex and region of residence. The HSE-PCRS pharmacy claims database does not contain clinical information regarding diagnosis, clinical outcome or over the counter (OTC) medication which may be obtained without a prescription [30]. Permission to use this database for research purposes was obtained from the HSE – PCRS.

The study population consisted of elderly individuals aged ≥ 65 years who were eligible for inclusion in the GMS scheme and who had received medication documented in the HSE-PCRS database during the study period. The study population was categorised by sex and subdivided into three age groups; 65–69, 70–74 and ≥ 75 years. Individuals with T2DM were identified using the prescription of any oral anti-hyperglycaemic (OAH) agent (ATC, A10B) either prescribed alone or in combination with any type of insulin (ATC, A10A), as a proxy for disease diagnosis. For the purposes of this paper the study population identified as having received medication for T2DM is referred to as the T2DM group. Individuals who did not receive any OAH agents during the study period were used as the comparator group for the analysis, and are referred to as the non T2DM group.

The burden of pharmacologically treated comorbidity in both the T2DM and the non T2DM group was ascertained using a modified version of RxRiskV index. The RxRiskV is a previously validated pharmaceutical based comorbidity index which is calculated from the sum of 45 potential disease groups derived from prescribing data using ATC classification codes [31]. The RxRiskV index was adapted for the purposes of the current study to include updated ATC codes for medications currently licensed in Ireland. Appendix 1 lists the medications and ATC codes used in the current study. In its original form, individuals were classified as having one of the conditions listed in the RxRiskV index if they had received at least one prescription filled for a disease class during a given study period [31]. This was modified in the present study to reflect the chronic nature of disease categories listed in the RxRiskV index. In the present study individuals were assumed to have one of the diseases if they received at least three consecutive prescriptions of a medication representing a specific disease class. In order to include the maximum number of elderly individuals fitting these criteria the study period was extended to sixteen months to include the last two months of 2009 and the first two months of 2011. Diabetes was excluded from the modified version of the RxRisk V index as it was the disease of interest. This modified version of the RxRisk V index is referred to as RxRiskV (mod). Ethical approval for this study was not required as the analysis for this study was carried out on an anonymised database.

### Data analysis

The comorbidity score was determined by calculating the maximum RxRiskV (mod) score for each individual and then grouping them according to their T2DM status. As the proportion of the study population with more than 10 conditions was small (0.78%) individuals with ≥ 10 were grouped together into a single category. The median comorbidity level, interquartile range and the prevalence of the most common comorbid conditions defined by the RxRiskV (mod) was calculated in those with and without T2DM. The association between T2DM and individual conditions in the RxRiskV (mod) index was assessed using the *Χ*^2^ test. The median number of comorbid conditions in the T2DM group was chosen as a proxy measurement to define low (<median) versus high (> = median) comorbidity. The association between T2DM and low versus high comorbidity was subsequently examined using the *X*^2^ test. Logistic regression analysis was performed to examine the relationship between the comorbid conditions and T2DM and adjusted for age (ref: 65–69 years) and sex (ref: female). The resulting associations are displayed as adjusted odds ratios with 95% confidence intervals (OR, 95%CI).

The total annual ingredient cost was calculated by summing the medication cost for each category included in the RxRiskV (mod) index for 2010. Bootstrapping was used to allow the comparison of means given the skewness of the cost data and replicated 2000 times so as to attain the 95% confidence interval [32]. The cost data were subsequently stratified by age, sex, T2DM status and number of comorbid conditions. SAS version 9.1 was used for the data analysis. Statistical significance at p <0.05 was assumed.

## Results

During January to December 2010, 445180 individuals aged ≥ 65 received at least three consecutive prescriptions recorded in the HSE-PCRS pharmacy claims database. Forty three per cent (191690 individuals) of the sample population were male. Half (50.3%) of the individuals were ≥ 75 years, 26.9% were 70–74 years and 22.8% were aged 65–69 years. In the study population 43165 individuals received three or more consecutive prescriptions for OAH agents. This represents a prevalence of 9.7% for T2DM in this elderly population. The prevalence of T2DM in this population was significantly higher in males (12.1%) compared to females (7.9%) (p < 0.0001). The prevalence of T2DM was highest in the middle age category 70–74 years, (10.1%) and lowest in the oldest study population ≥75 years (9.4%) (p < 0.0001).

The median number of comorbid conditions was found to be significantly higher in the group with T2DM (5 conditions, IQR 3–6) compared to the non T2DM group (3 conditions, IQR 1–5). The association between low versus high comorbidity was significant (*X*^2^ =16355, df =10, p < 0.0001). The odds of having ≥ 5 comorbid conditions was almost three times higher in the T2DM group compared with the non T2DM group (OR = 2.82, 95% CI = 2.76-2.88, p < 0.0001). In the study population with T2DM, females in the youngest (65–69 years) and middle (70–74 years) age categories had a higher median number of comorbid conditions when compared to males (5 vs. 4 conditions) (Table 1). In the non T2DM group there was no significant difference in the median number of comorbid conditions when stratified by age group and sex (Table 1).

**Table 1:** Median number of comorbid conditions stratified by age, sex and T2DM status (Total n = 445,180)

***Sex***	***Age***	***T2DM group (n 43165)***	***T2DM median no. of comorbid conditions [IQR]***	***Non T2DM group (n 402015)***	***Control median no. of comorbid conditions [IQR]***
**Male**					
	65 - 69	5535	4 [3 – 6]	38606	3 [1 – 4]
	70 - 74	6948	4 [3 – 6]	50023	3 [1 – 5]
	≥75	10690	5 [3 – 6]	79888	4 [2 – 5]
**Female**					
	65 - 69	4610	5 [3 – 6]	52770	3 [1 – 4]
	70 - 74	5109	5 [4 – 6]	57726	3 [1 – 5]
	≥75	10273	5 [4 – 7]	123002	4 [2 – 6]

The prevalence of 35 of the 45 conditions listed in the RxRiskV (mod) index was significantly higher in the group with T2DM than in the non T2DM group. The results from the logistic regression analysis on individual comorbid conditions included in the RxRiskV (mod) index with a prevalence of ≥ 10% in the study population showed an increased likelihood of individuals with T2DM having received a prescription for these conditions. The prevalence of co-prescribing and the odds ratios adjusted for age and sex are summarised in Table 2*.*

**Table 2:** Chronic medical conditions included in the RxRiskV (mod) index with ≥10% prevalence in the study population

***RxRiskV (mod) category***	***Non T2DM (%)***	***T2DM (%)***	Adjusted Odds Ratio **	***95% CI***
**Concordant conditions/therapies**				
**Cardiovascular system**				
Hyperlipidemia	42.6	78.5	4.95	4.83 - 5.06
Anti-platelet agents*	39.8	71.7	3.84	3.76 - 3.93
Heart disease	39.4	61.4	2.44	2.39 - 2.49
Hypertension	21.7	32.2	1.79	1.75 - 1.83
**Discordant conditions/therapies**				
**Digestive system**				
Gastric reflux and peptic ulcer	34.5	46.4	1.67	1.63 - 1.70
**Mental health**				
Depression	16.0	20.5	1.46	1.43 - 1.50
**Respiratory system**				
Chronic airway disease	14.4	18.6	1.35	1.32 - 1.40
**Musculoskeletal**				
Osteoporosis	14.3	9.3	0.73	0.70 - 0.75
**Pain management**				
Anti-inflammatory agents	13.0	14.1	1.14	1.11 - 1.18
Pain (Opiates)	10.0	12.9	1.42	1.38 - 1.47

* Non specific marker for cardiovascular disease.

** adjusted for age and sex.

The majority of comorbidity in the group with T2DM related to concordant conditions or therapies associated with the cardiovascular system (CVS). A number of discordant conditions were also significantly co-prescribed more often in the group with T2DM (p < 0.0001). The most prevalent of these were gastric reflux and peptic ulcer, depression and chronic airway disease. The prevalence of co-prescribed medication for osteoporosis was, however, significantly lower in the group with T2DM (OR = 0.73, 95%CI = 0.70-0.75, p < 0.0001) when compared to the control group.

The mean annual ingredient cost of comorbidity in the study population with T2DM was higher (€1238.67, 95% CI = €1238.20 - €1239.14) than for the control group (€799.28, 95% CI = €799.14 - € 799.41). When the cost data was stratified by age and sex, a similar significant difference between groups was observed in both men and women with the mean ingredient cost of comorbidity increasing with increasing age (Table 3). The mean drug ingredient cost of comorbidity was higher in women compared to men across all age categories in the study population with T2DM, and was also higher in women in the middle (70–74 years) and oldest (≥ 75 years) age categories in the study population without T2DM (Table 3). Further analysis of the cost data, stratified by number of comorbid conditions, found that the mean annual ingredient cost of chronic comorbidity was higher in the non T2DM group compared to the group with T2DM in patients with a low number (≤ 2) of comorbid conditions. Conversely, in the study population with a high number (≥ 4) of comorbid conditions, the mean annual ingredient cost was higher in the T2DM group compared to the group without T2DM. There was no difference in the mean annual ingredient cost between the two groups in patients with three comorbid conditions. These results are summarised in Table 3.

**Table 3:** Mean annual ingredient cost of chronic comorbidity in the study population with and without T2DM

***Sex***	***Age (yrs)***	***T2DM group***	***Non T2DM group***	Cost ratio*
**Male**				
	65-69	**1183. 43** [1182.12, 1184.75]	**683. 85** [683.41, 684.28]	1.73
	70-74	**1184. 35** [1183.22, 1185.47]	**744. 02** [743.67, 744.37]	1.59
	≥75	**1245. 51** [1244.65, 1246.36]	**871. 33** [871.02, 871.65]	1.45
**Female**				
	65-69	**1223. 89** [1222.43, 1225.31]	**667.52** [667.16, 667.88]	1.83
	70-74	**1293. 06** [1291.74, 1294.38]	**764.89** [764.57, 765.22]	1.69
	≥75	**1279. 10** [1278.05, 1280.14]	**882.95** [882.71, 883.18]	1.45
**No. of comorbid conditions**				
**0**		**0**	**0**	1
**1**		**193. 45** [192.82, 194.08]	**225.02** [224.86, 225.17]	0.86
**2**		**386. 59** [385.99, 387.20]	**414.52** [414.34, 414.69]	0.93
**3**		**628. 65** [628.10, 629.21]	**628.35** [628.15, 628.54]	1
**4**		**897. 08** [896.42, 897.74]	**874.86** [874.60, 875.11]	1.03
**5**		**1186. 88** [1186.14, 1187.62]	**1153.70** [1153.38, 1154.02]	1.03
**6**		**1485. 14** [1484.16, 1486.13]	**1454.08** [1153.38, 1154.02]	1.02
**7**		**1807.74** [1806.40, 1809.08]	**1764.68** [1764.05, 1765.31]	1.02
**8**		**2130. 86** [2129.05, 2132.67]	**2078.91** [2078.03, 2079.79]	1.03
**9**		**2461. 44** [2458.71, 2464.17]	**2436.44** [2435.00, 2437.87]	1.01
**≥10**		**3151. 03** [3146.39, 3155.67]	**3023.80** [3022.13, 3025.46]	1.04

[95% Confidence Interval].

* T2DM group : non T2DM group.

## Discussion

This is the first large scale study using a national pharmacy claims database in Ireland to investigate the prevalence, type and ingredient cost of comorbidity present in the elderly GMS eligible population (≥ 65 years) with and without T2DM. It has also successfully modified the RxRiskV index to include the ATC codes of pharmacological agents currently licensed for use in Ireland and strengthened the definition of “chronic” used in the original version, by specifying that an individual must have received a minimum of three consecutive prescriptions for a disease class. The results show that during the study period the elderly population with T2DM had a higher level of comorbidity and associated drug costs when compared to the non T2DM group. These results are similar to the findings of previous studies that investigated comorbidity in diabetic populations in Finland and Australia [33,34].

Overall, cardiovascular-related concordant conditions accounted for a substantial proportion of the comorbidity in the study population both with and without T2DM. A higher level of cardiovascular-related comorbidity in patients with T2DM has been reported before [33,35] and reflects the established association between T2DM and conditions affecting the cardiovascular system [36]. The high rate of co-prescription of anti-platelet therapy, anti-hypertensive medication and cholesterol lowering agents in patients with T2DM also suggests prescriber adherence to the current Irish cardiovascular health policy which advocates active and aggressive management of cardiovascular risk factors in individuals with diabetes [37].

The most prevalent discordant comorbid conditions in the current study were gastric reflux/peptic ulcer, depression and chronic airway disease. This is consistent with the findings of a previous Australian study that used the RxRiskV index to examine the level of comorbidity in a cohort of veterans with T2DM [34]. It is possible however, that the higher frequency of co-prescribing treatment for gastric reflux/peptic ulcer in the group with T2DM may reflect the use of proton pump inhibitors (PPIs) as a gastroprotective agent in patients taking the OAH agents rather than the presence of gastrointestinal morbidity.

Previous studies that have investigated comorbidity in populations with diabetes have also reported an increase in the prevalence of depression [38,39]. The frequency of co-prescription of anti-depressant medication in the current study was significantly higher in the group with T2DM. There is conflicting evidence as to whether there may be a physiological basis for the observed association between diabetes and depression or whether it is due to psychosocial stress associated with having a chronic condition [40]. There is evidence, however, to suggest that the presence of comorbid depression in individuals with diabetes is associated with poorer medication adherence [41] and an increased risk of diabetes related complications [42]. It is imperative therefore, that depression in patients with T2DM is recognised and treated given the adverse outcomes associated with such comorbidity.

Osteoporosis was the only condition included in the RxRiskV (mod) index with a prevalence of ≥10% in the study population as a whole that was prescribed for less frequently in the group with T2DM. This finding is perhaps unexpected as a recent meta-analysis demonstrated that individuals with diabetes have an increased risk of various types of bone fracture [43]. It is possible that the result of the present study may infer an inadequacy in the level of prescribing anti-osteoporotic medication to elderly individuals with T2DM.

Research has shown that elderly patients with multiple unrelated medical conditions may be undertreated [44]. Health professionals attending to patients with chronic conditions must remain vigilant for other disorders to ensure they are treated appropriately [44]. This is particularly important, considering information relating to the care of patients with multiple conditions is scarce [5]. Evidence based guidelines established for the treatment of diabetes and other major chronic conditions have focused too narrowly on the management of single conditions [9] and may not be appropriate for patients with comorbidity unrelated to the index condition. This should be taken into consideration when proposing new health strategies. Policy makers should alter the focus of initiatives away from individual diseases towards policies that reflect the holistic requirements of individuals with multiple conditions [7,45].

The economic liability posed by diabetes has been discussed extensively in the published literature [22]. It has been well documented that the treatment of complications associated with diabetes account for the majority of the economic cost associated with patient care [22]. The cost analysis presented in the current study focuses solely on the ingredient cost of prescription drugs and did not include other expenses such as health care utilisation or patient out-of-pocket expenses. The results of the current analysis suggest that the treatment of comorbid conditions (both concordant and discordant) pose a significant additional annual ingredient cost in the GMS eligible elderly population with T2DM compared to the study population without the condition. It would, therefore, be advisable that both types of comorbidity be taken into consideration in future economic evaluations of costs associated with diabetes.

The results of this study have important clinical implications for both patients and health professionals. There are also substantial economic implications for decision makers responsible for providing the most cost-effective health care. The results of the current study indicate that those with T2DM have greater number of comorbid conditions, both related and unrelated to diabetes, and that these are associated substantial increased cost. Increased education and earlier intervention programmes for patients with diabetes are needed so as to avoid the costly consequences of poor adherence and management of their condition. A structured management care programme provided in general practice, in conjunction with a multi-disciplinary team of health professionals; with direct and immediate access to specialist services as required, would facilitate this greatly. In Ireland, this type of programme has been implemented in the Midlands health region for patients with diabetes and has produced encouraging results [46]. The nationwide implementation of this type of programme could yield an overall improvement in patient management and costs associated with diabetes.

The present study has a number of limitations. The HSE-PCRS pharmacy claims database upon which the data for this study was based does not contain clinical diagnoses. As a result patients with T2DM were identified in study population using the prescription of any OAH agents, with or without insulin, as a proxy for disease diagnosis. This definition was unable to take into account patients with T2DM who were treated using diet alone. In spite of this methodological limitation it is likely that the present study has captured the majority of diagnosed T2DM in the study population, given the results of a previous community based study that reported that the majority of patients (74%) with T2DM were treated with OAH agents [47]. In addition, the current study was unable to take into account patients with medication treated T2DM who did not meet the eligibility criteria for the GMS scheme. It is possible that non medical card patients may have received their diabetes medication through other community drug schemes such as the Long Term Illness (LTI) scheme or Drugs Payment Scheme (DPS) or paid for their medications privately. In 2010, medical card holders represented a very high proportion of the Irish elderly population ≥ 70 years (98.4%). It is, therefore, likely that the results of the present study represent an accurate account of medication treated T2DM, comorbidity and the associated ingredient cost in this age group. For the age group 65–69 years, only half are eligible for the GMS scheme, which may represent a slightly more deprived and sicker population. Previous research has indicated that medical card holders on average visit their doctor more frequently per year [48] and have poorer health [49] when compared to non medical card holders. There are limitations to using the bootstrapping methods including the assumption that the distribution of the data from the sample is a reasonable estimate of the population distribution from which it came. Given the very large sample size and the high percentage of the population captured this is unlikely to be a major source of bias. There may also have been some sampling error in the selection of random samples in the bootstrap procedure, but with 2000 samples chosen this is unlikely. Finally, a medication listed for one disease category in the RxRiskV (mod) may have a number of licensed indications for use. In order to limit the effect of this in the present study, medications with more than one indication were assigned to a single disease category which reflected the highest ranked licensed indication.

## Conclusion

This study has shown that older patients with T2DM in Ireland have a greater prevalence of comorbidity when compared to those without the condition. It has also shown increased economic cost in terms of drug expenditure for both concordant and discordant comorbidity in this study population. These findings highlight the need for health policy makers and economists to ensure that both concordant and discordant comorbid conditions are taken into account when planning for future health care needs of those with diabetes.

## Appendix I

List of ATC codes used in the RxRiskV (mod)

**Alcohol dependency** [N07BB03, N07BB04, N07BB01]

**Allergies** [R01AC, R01AD, R06AD02, R06AD03, R06AD04, R06AD05, R06AD06, R06AD07, R06AD08, R06AD09, R06AD52, R06AD55, R06AE, R06AK, R06AX, ***Excluding*** R06AX27 R06AX28 R06AX53 R06AX58]

**Anti-coagulation therapy** [B01AA03 B01AA04, B01AA07 - B01AA11, B01AB01, B01AB02, B01AB04 - B01AB06, B01AB10]

**Anti-platelet therapy** [B01AC04 - B01AC19, B01AC30, B01AC22, B01AC23]

**Anxiety** [N05BA01 - N05BA12, N05BB01]

**Arrhythmia** [C01AA05, C01BA01-C01BD01]

**Angina** [C01DA02, C01DA04, C01DA05, C01DA07, C01DA08, C01DA09,

C01DA13, C01DA14, C01DX16, C01EB15, C01EB17, C01EB18]

**Benign prostate hypertrophy** [G04CA02-G04CA03]

**Bipolar disorder** [N05AN01]

**Chronic Heart failure** - ***Must have both loop diuretic*** [C03CA, C03CB, C03CC01, C03DA] ***and ace inhibit*** [C09AA01, C09AA02, C09AA03, C09AA04, C09AA05, C09AA06, C09AA07, C09AA08, C09AA09, C09AA10, C09CA06, C09CA07, C09CA01, C09CA03]

**Dementia** [N06DA02, N06DA03, N06DA04, N06DX]

**Depression** [N06A]

**End stage renal disease** [B03XA, V03AE02, V03AE03, and A11CC]

**Epilepsy** [N03AA01-N03AA04, N03AA30, N03AB01-N03AB05 N03AB52,

N03AB54, N03AC01, N03AC02, N03AC03, N03AD01, N03AD02, N03AD03, N03AD51, N03AE01, N03AF01, N03AF02, N03AG01, N03AG02, N03AG03, N03AG04, N03AG05, N03AG06, N03AX]

**Gastric-oesophageal reflux disorder & Peptic ulcer** [A02B]

**Glaucoma** [S01EA01, S01EA02, S01EA03, S01EA04, S01EA05, S01EA51”

S01EB01, S01EB02, S01EB03, S01EC03, S01EC04, S01ED01, S01ED02, S01ED03, S01ED04, S01ED05, S01ED06, S01ED51, S01ED52, S01ED54, S01EE01, S01EE02, S01EE03, S01EE04, S01EX01, S01EX02]

**Gout** [M04AA01, M04AA02, M04AA03, M04AA51, M04AB01, M04AB02, M04AB03, M04AB04, M04AC01]

**Hepatitis C** [J05AB54]

**HIV** [J05AE01 - J05AE08, J05AF01 - J05AF11, J05AG01, J05AG02, J05AG03 J05AR01-J05AR06, J05AX07]

**Hyperkalaemia** [V03AE01]

**Hyperlipidemia** [C10AA, C10AB, C10AC, C10AD, C10AX, C10BA, C10BX]

**Hypertension** [C03AA, C03AB, C03AH, C03AX01, C02CA04”

C03BA02, C03BA03, C03BA04, C03BA05, C03BA07, C03BA08, C03BA09, C03BA10, C03BA11, C03DB01, C03DB02, C03EA, C09BA02, C09BA03, C09BA04, C09BA05, C09BA06, C09BA07, C09BA08, C09BA09,

C09BB, C09DB, C09DA02, C09DA03, C09DA04, C09DA06, C09DA07,

C09DA01, C02AB01, C02AB02, C02AC01, C02AC02, C02AC04, C02AC05, C02DB02, C02DB03, C02DB04, C02DC01, C02DD01, C02DG01, C02KA01, C02KB01, C02KC01, C02KD01, C02KX01, C09XA]

**Hypothyroidism** [H03AA01, H03AA02].

**Heart disease** [C07AA01, C07AA02, C07AA03, C07AA05, C07AA06,

C07AA07, C07AA12, C07AA14, C07AA15, C07AA16, C07AA17, C07AA19,

C07AA23, C07AA27, C07AA57, C07AB, C07AG01, C07AG02, C07BA02, C07BA05, C07BA06, C07BA07, C07BA12, C07BA68, C07BB02, C07BB03, C07BB04, C07BB06, C07BB07, C07BB52, C07BG01, C07CA02, C07CA03, C07CA17, C07CA23, C07CB02, C07CB03, C07CB53, C07CG01, C07DA06, C07DB01, C07FA05, C07FB02, C07FB03, C07FB07, C08CA01, C08CA02, C08CA03, C08CA04, C08CA05, C08CA06, C08CA07, C08CA08, C08CA09, C08CA10, C08CA11, C08CA12, C08CA13, C08CA14, C08CA15, C08CA55,

C08CX01, C08DA01, C08DA02, C08DA51, C08DB01]

**Inflammatory bowel disease** [A07EC01, A07EC02, A07EC03, A07EC04]

**Liver failure** [A06AD11]

**Malignancies** [L01AA01, L01AA02, L01AA03, L01AA05, L01AA06,

L01AA07, L01AA08, L01AB, L01AC, L01AD, L01AG01, L01AX

L01BA01, L01BA03, L01BA04, L01BB02, L01BB03, L01BB04, L01BB05, L01BB06, L01BB07, L01BC, L01CA, L01CB, L01CC01, L01CD L01CX01 L01DA01

L01DB, L01DC, L01XA, L01XB01, L01XC, L01XD01, L01XD03, L01XD04 L01XD05 L01XD06 L01XE, L01XX, L02BA01, L02BA02, L02BG02, L02BG03, L02BG04, L02BG06, L02BB01, L02BB03, L02AE02, L02AE04, L02AB01]

**Migraine** [N02CA01, N02CA02, N02CA04, N02CA07, N02CA51

N02CA52, N02CA72 N02CB01, N02CC01, N02CC02, N02CC03, N02CC04, N02CC05, N02CC06, N02CC07, N02CX01

**Osteoporosis** [M05BA, M05BB, M05BX03, G03XC01, A12AX92]

**Pain - Opiates** [N02AA, N02AB, N02AC01, N02AC03, N02AC04, N02AC05, N02AC52, N02AC54, N02AC74, N02AD01, N02AD02, N02AE01, N02AF01,

N02AF02, N02AG, N02AX01, N02AX02, N02AX52, NO2AX05]

**Pain - Anti-inflammatory agents** [M01AB, M01AC01, M01AC02, M01AC04, M01AC05, M01AC06, M01AE, M01AG, M01AH, N02BE51, NO2BA01, N02BG06]

**Pancreatic insufficiency** [A09AA02]

**Parkinson’s disease** [N04AA01, N04AA02, N04AA03, N04AA04, N04AA05,

N04AA08, N04AA09, N04AA10, N04AA11, N04AA12, N04AB01, N04AB02, N04AC01, N04AC30, N04BA01, N04BA02, N04BA03, N04BA04, N04BA05, N04BA06, N04BB01, N04BC01, N04BC02, N04BC03, N04BC04, N04BC05, N04BC06, N04BC07, N04BD01, N04BX01, N04BX02, N0BC09, N04BD02]

**Psoriasis** [D05BB01, D05BB02, D05AX]

**Psychotic illness** [N05AA, N05AB, N05AC, N05AD, N05AE, N05AF, N05AG

N05AH, N05AL, N05AN01, N05AX]

**Chronic airways disease** [R03AC, R03AK, R03BA, R03AB,

R03BC01, R03BC03, R03BX01, R03CA02, R03CB, R03CC, R03CC53, R03DA, R03DB, R03DC, R03BB]

**Smoking cessation** [N07BA01, N07BA03]

**Steroid responsive disease -*Systemic corticosteroid use*** [H02AB, H02AA]

**Transplant** [L04AA01, L04AA02, L04AA03, L04AA04, L04AA05,

L04AA06, L04AA08, L04AA09, L04AA10, L04AA11, L04AA12,

L04AA14, L04AA15, L04AA16, L04AA17, L04AA18, L04AA19,

L04AA21, L04AD02, L04AX01]

**Tuberculosis** [J04AB04, J04AB05, J04AB30, J04AC01, J04AC51, J04AD01, J04AD02, J04AD03, J04AK01, J04AK02]

**Neurogenic Bladder and Urinary Incontinence** [V07AN]

**Ostomy** [V07AS]

## Abbreviations

(T2DM): Type 2 diabetes; (OAH): Oral anti-hyperglycaemic; (HSE-PCRS): Health Service Executive – Primary Care Reimbursement Services; (ATC): Anatomical Therapeutic Chemical; (GMS): General Medical Scheme; (OTC): Over-the-counter; (LTI): Long Term Illness Scheme; (DPS): Drugs Payment Scheme.

## Competing interests

The authors declare that they have no competing interests.

## Authors’ contributions

All authors jointly developed the concept of the study. MT assisted with the development of the RxRiskV (mod). KB gave statistical advice for the study. MO’S conducted the statistical analysis. MO’S wrote the initial draft of the manuscript. KB and MT revised the manuscript. All authors revised and approved the manuscript prior to submission.

## Pre-publication history

The pre-publication history for this paper can be accessed here:

http://www.biomedcentral.com/1472-6963/13/23/prepub
